# Predictors for Burnout Among Healthcare Workers in a Post -Covid Era

**DOI:** 10.1192/j.eurpsy.2024.1035

**Published:** 2024-08-27

**Authors:** L. Lim, G. Heng, L. Loh, Y. H. Chan, L. Eng, C. Chan, J. Fam

**Affiliations:** ^1^Psychiatry; ^2^Department of Psychiatry, Singapore General Hospital; ^3^ Biostatistics Unit, National University of Singapore, Yong Loo Lin School of Medicine; ^4^Duke- NUS Medical School, Singapore, Singapore

## Abstract

**Introduction:**

We aimed to study predictive factors for burnout (BO) among healthcare workers in a tertiary hospital in Singapore.

**Objectives:**

We hypothesized that burnout would be assoiciated with singles, females, and foreign born staff recently moved into this country, unaccompanied by family members.

We further hypothesised that BO would be associated with those scoring less on resilience. Recognising that social support mitigated against stress and burnout, we hypothesized that those who perceived less support would be more prone to BO.

**Methods:**

The study questionnaire was sent via corporate email to all staff with email access. We stressed that data would be fully anonymised. No financial rewards were given for participation which was carried out on a voluntary basis.

The following instruments were used, viz. F-SozU K-6, a brief form of the perceived social support questionnaire; Connor Davidson Resilience Scale; Oldenburg Burnout Inventory; Patient Health Questionnaire-4 item; Demand Control Support Questionnaire and Leisure Time Satisfaction Scale. Ethics approval for the study was sought from the SingHealth Centralised Institutional Review Board, which granted exemption of participant consent.

Analyses were performed using Stata version 17.0 (StataCorp. 2021), with statistical significance set as 2-sided 5% (p<0.05). The reliability and internal consistency of the scales used were assessed using Cronbach Alphas and Confirmatory Factor Analysis (CFA).

**Results:**

Neither males nor females were more at risk for BO. And contrary to what we hypothesised those who recently moved to this nation were not at greater risk for BO (p>0.05). Multivariate analyses showed that younger workers displayed higher burnout scores (p < 0.001).The psychological demand sub-score was positively associated with burnout [ 0.61 (95% CI 0.45 to 0.77), p < 0.001)]. Conversely, decision latitude [-0.33 (95% CI -0.44 to -0.21), p < 0.001)] and support [-0.47 (95% CI -0.60 to -0.35), p < 0.001] were negatively associated with BO.

Those who experienced anxiety or depressive symptoms were respectively more likely to experience burnout [0.30 (95% CI 0.02 to 0.58), p = 0.035 and 0.72 (95% CI 0.41 to 1.02), p < 0.001], with a clear association between higher PHQ-4 scores and risk for burnout (r = 0.619).

Moreover, satisfaction with utilisation of leisure time was inversely related to BO [-0.55 (95% CI -0.68 to –0.41; p < 0.001)]. We could not find any association between number of years worked, profession, marital status and perceived social support and BO, on multivariate anbalysis (p>0.05).

**Conclusions:**

Strress reduction interventions should be made available for all staff, especially addressing those at highest risk for burnout.

**Disclosure of Interest:**

None Declared

